# Research understanding, attitude and awareness towards biobanking: a survey among Italian twin participants to a genetic epidemiological study

**DOI:** 10.1186/1472-6939-10-4

**Published:** 2009-06-16

**Authors:** Virgilia Toccaceli, Corrado Fagnani, Lorenza Nisticò, Cristina D'Ippolito, Lorenzo Giannantonio, Sonia Brescianini, Maria Antonietta Stazi

**Affiliations:** 1 Genetic Epidemiology Unit, National Centre of Epidemiology, Surveillance and Health Promotion, Istituto Superiore di Sanità, V.le Regina Elena 299, Rome 00161, Italy

## Abstract

**Background:**

The Italian Twin Registry (ITR) has been carrying out several genetic-epidemiological studies. Collection and storage of biological material from study participants has recently increased in the light of biobanking development. Within this scenario, we aimed at investigating understanding, awareness and attitude towards blood/DNA donation of research participants. About these quite unknown dimensions more knowledge is needed from ethical and social perspectives.

**Methods:**

Cross-sectional mail survey to explore three dimensions: (i) understanding of aims and method of a specific study, (ii) attitude (three ideas for donation: "moral duty", "pragmatism", "spontaneity") and (iii) awareness (i.e. the recall of having been asked to donate) towards blood/DNA donation for research, among all the Italian twins who had participated in Euroclot (n = 181), a large international genetic-epidemiological study. Multivariate models were applied to investigate the association of sex, age, education and modality of Euroclot recruitment (twins enrolled in the ITR and volunteers) with the targeted dimensions. Pair-wise twin concordance for the "pragmatic" attitude was estimated in monozygotic and dizygotic pairs.

**Results:**

Response rate was 56% (99 subjects); 75.8% understood the Euroclot method, only 33.3% correctly answered about the study aim. A significantly better understanding of aim and method was detected in "volunteers". Graduated subjects were more likely to understand study aim. In the overall sample, the "pragmatic" attitude to blood donation reached 76.8%, and biobanking awareness 89.9%. The latter was significantly higher among women. Monozygotic twins were more concordant than dizygotic twins for the "pragmatic" attitude towards blood/DNA donation for research.

**Conclusion:**

Level of understanding of aims and methods of a specific research project seems to vary in relation to modalities of approaching research; most of the twins are well aware of having been asked to donate blood for biobanking activities, and seem to be motivated by a "pragmatic" attitude to blood/DNA donation. Genetic influences on this attitude were suggested. The framing of interests and concerns of healthy participants to genetic-epidemiological studies should be further pursued, since research, particularly for "common diseases", is increasingly relying on population surveys and biobanking.

## Background

Research understanding as well as awareness and attitude of study participants towards blood/DNA donation for research are quite unknown fields, especially for what concerns the Italian research.

Although genetic epidemiology in the field of common diseases seldom faces with harmful procedures or results to be considered like "verdicts", it is always unethical to treat participants as mere "means" [1]. Making individuals aware of the aims of the study they are entering and making them participate in a balanced relationship "researcher-subject of research" are fundamental actions that go beyond the Ethical Board reviewing process of the protocols. Moreover, healthy individuals, who are required for epidemiological population-based studies, generally do not have urgent interests or immediate advantages to be obtained from these studies; actions which promote understanding of the aims and methods and enhance awareness of biobanking activities may contribute, on different perspectives, to increase individual trust and willingness to participate in research [2].

Within this scenario, the understanding of a specific study is worth being assessed among participants as a starting point. Moreover, research based on stored blood and DNA has been gaining a rapidly increasing relevance [3], and the impact of the biobank tool on research advancements in the medium and long term cannot be quickly assessed; yet, the benefits of this tool for faster, cheaper and better research in terms of data quality and statistical power can easily be envisaged. The Italian Twin Registry is a population-based Registry, currently involved in several genetic-epidemiological studies, which require population samples of twins to be carried out; these studies also need biological material and DNA to be analysed as well as to be stored for further use. It is our opinion that donors generally have to give high level trust to those who manage biobanks and to the uses each biobank is dedicated to. In our country, a comprehensive law regulating biobanking has not yet been issued, and at international level it has been often recognized that the act of withdrawing is the only "active role" of participants in human genetic biobanks [4]. In this framework, motivation and attitude towards biobank-based research as well as donors' awareness for donation are relevant issues to be investigated from both scientific and ethical perspectives [5].

The aim of the present survey is to explore, among a sample of participants to a genetic-epidemiological study, understanding of aim and method as well as motivation and awareness towards DNA donation for research biobanking.

## Methods

### The survey and the questionnaire

Within the research activities promoted by the Italian Twin Registry (ITR) [6], a cross-sectional survey by mail questionnaire was conducted on all the Italian twins who had participated to Euroclot, a large international genetic-epidemiological study aimed at identifying genes associated with variations of the end-stage clotting process, and their role in the pathogenesis of stroke .

**Figure 1:**
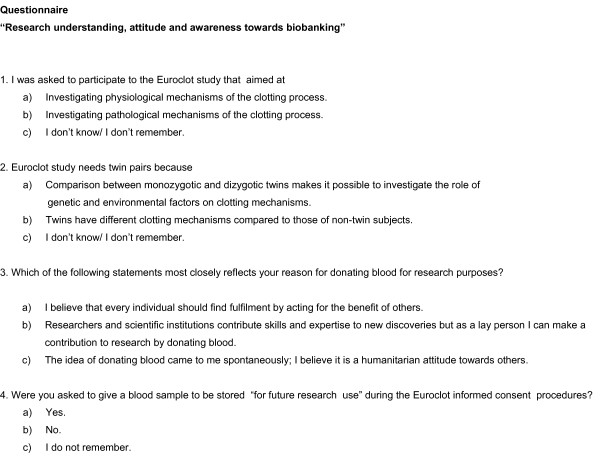
**Questionnaire "Research understanding, attitudes and awareness towards biobanking"**.

A questionnaire (Fig. 1) was developed to investigate three dimensions: (i) participants' "understanding" of Euroclot aim and method, (ii) their "attitude" and (iii) "awareness" towards blood donation for research.

(i) Understanding was addressed by two questions about Euroclot aim and method (Fig. 1, items n.1, 2). Response categories referred to the explanations provided in the letter sent for recruitment as well as during the informed consent procedure to the Euroclot participants. In these two occasions the main elements describing aims and methods were provided along with the key terminology reported later in the survey questionnaire (e.g. "physiological" and "pathological" mechanisms, "genetic factors", "environmental factors", "comparison of monozygotic vs dizygotic twins", "clotting process"). As several Euroclot participants thought that twins had a "different physiology" compared to singletons, this idea was used to build the "incorrect" answer choice for the question on method.

(ii) The questionnaire also explored motivations which might lie behind the individual choice to donate a blood sample for the ITR biobank [7]. Three main ideas, drawn from different ethical and anthropological approaches to interpret human behaviour, were conceptualized as motivation and were used for response categories to question n. 3 (Fig. 1, item n. 3). One idea highlights a moral duty to donation for research where the individual believes he/she should find fulfilment by acting for the benefit of others [8]. A second idea relies on a kind of pragmatic attitude where donation is interpreted as a contribution to research advancements in a balanced relationship between participants and researchers [9]. A third idea focuses on spontaneity of donation as an inner attitude of the human beings not culturally driven [10]. The categorization process was also guided by the results of a series of pre-interviews conducted on a subsample of Euroclot participants. Open responses were avoided to skip possible misinterpretations and errors in categorization.

(iii) The last question (Fig. 1, item n. 4) regarded attention and awareness towards blood donation for biobanking. As all the Euroclot participants had agreed to donate additional blood, we considered the recall to have been asked to donate as a proxy for awareness. "*I do not know/remember*" was included for "understanding" and "awareness" questions (Fig. 1, items n.1, 2 and 4) to avoid that individuals might guess the "correct answer" introducing a bias; the "*I do not know/remember*" responses were counted as "non-correct" answers.

Other characteristics recorded in the questionnaire were age, educational level, and twin zygosity (assessed by a standard questionnaire regarding physical similarity of the twins) [11]. A final version of the questionnaire was obtained after it was tested on a core of twins (12 pairs) randomly chosen among the Euroclot participants.

### Survey Population

We chose to carry out this survey on the Euroclot study participants because, for this research, a thorough person-to-person interaction was performed during information and consent procedures.

In fact, previous investigations on the effects of different interventions to increase participants' understanding, even if almost exclusively on clinical trials, showed that person-to-person interactions between participant and researcher may be more effective than any other method [12].

The Italian Euroclot participants were 181 subjects (89 twin pairs and one triplet). Recruitment for the Euroclot study was performed in February-April 2006 with the following procedure: 135 individuals (age range: 21–24; mean age: 22.7), previously enrolled in the ITR ("ITR-enrolled"), agreed to participate after having received an informative leaflet by mail and having been subsequently contacted by telephone. Further 46 participants (age range: 21–63; mean age: 43.3), not enrolled in the ITR ("volunteers"), asked to participate to Euroclot as a result of the local press and radio advertising campaigns. The Euroclot study had received approval by the Ethical Review Board of the Istituto Superiore di Sanità (Rome, Italy) and Euroclot examinations were conducted from April to October 2006.

Both the "ITR-enrolled" and "volunteer" subjects were asked about family history of stroke and other cardiovascular diseases: no significant differences emerged between the two groups.

In November 2006, the survey questionnaire was sent by mail to the whole sample of Euroclot participants; responses were received up to June 2007.

### Ethical procedures for the Euroclot study

All the information about different aspects of the Euroclot study, i.e. aims, twin methodology, clinical/non clinical value of results, donation of blood samples for ITR Biobank, rights of participants/donors including access and withdrawal were illustrated during a face-to face conversation, before the health examination, by two medical doctors and one bioethics adviser. All subjects were explained they would have no immediate benefit from blood donation for future undetermined research, and that they would not be discriminated in case of refusal or withdrawal. It was also clearly explained that the consent regarded exclusively the storage of samples in the biobank and the recording of related data, and not the future re-use of samples and data. Specifically, in agreement with the recent Italian regulation on "Genetic Data Treatment" [13], as a highly conservative option, we structured the consent on the basis that donors would be re-contacted to authorize any further use of their samples (i.e. a new study-specific consent for any single use of the biological material and data). Legal requirements were accomplished to guarantee privacy and confidentiality according to the Italian Law on Personal Data Treatment [14].

### Statistics

Frequency distributions of replies were computed.

Multiple logistic regression models were done to evaluate the independent association of gender, age, education, time elapsed between Euroclot examinations and survey responses, and modality of twin recruitment ("ITR-enrolled" and "volunteer" subjects) with each of the investigated dimensions. Response items were dichotomously recoded: for item 1 and 2 (understanding of Euroclot aims and methods), correct answers vs incorrect answers plus "*I don't know/remember*"; for item 3 (attitude), "pragmatic" answers (b response) vs other answers; for item 4 (awareness), "*yes*" answers vs "*no*" plus "*I don't know/remember*". Robust estimation of standard errors was used to take account of data clustering on twin pairs.

Pairwise twin concordance [C = n_c_/(n_c_+n_d_), n_c _= number of concordant pairs and n_d _= number of discordant pairs] for the "pragmatic" attitude to donation (item 3, b response) was estimated in MZ and DZ pairs separately, and interpreted under the assumptions of the classical twin method [15,16]: a higher concordance in MZ twins, genetically identical, than in DZ twins, who share on average 50% of their genes, suggests that genetic factors may influence the predisposition to the attitude. To this end, the dichotomous version of the item was used. The triplet was treated as three DZ pairs.

All analyses were performed using Stata software (Release 8, 2003).

## Results

### Survey response

Out of the overall 181 Euroclot participants who received the survey questionnaire, 101 (56%) replied. Two individuals (one twin pair) were excluded due to missing responses, leaving a total of 99 subjects, 26 males and 73 females. No significant differences were observed between respondents and non-respondents for sex (p = 0.15) and zygosity (p = 0.26). The age range of respondents was identical to that of the entire Italian Euroclot cohort. Median age of respondents was slightly higher compared to non-respondents (22.9 vs 22.5 years; p = 0.02).

Among the 135 "ITR-enrolled" twins, 61 replied (15 males and 46 females; response rate: 45%), while out of the 46 "volunteers", 38 replied (11 males and 27 females; response rate: 83%).

Frequency distributions of replies to the addressed dimensions are shown in Table 1.

[see Additional file 1]

### Understanding of aim and method of the Euroclot study

An apparent discrepancy emerged for understanding: while a fairly high proportion of individuals (75.8%) understood the Euroclot method, only 33.3% correctly answered about the study aim. The logistic regression model (Table 2) indicated a significantly higher level of understanding of both study aim (OR = 8.23; 95%CI: 1.28–52.81) and method (OR = 11.41; 95%CI: 1.10–118.38) in "volunteers" than in "ITR-enrolled" subjects. This last result was independent of age and also of time elapsed between Euroclot examination and survey response that were included in the model but, by themselves, were not associated with the probability of providing the correct answer. An increased level of understanding in more educated subjects was also suggested; in particular, for understanding of aim, the odds ratio was nine times higher for college (OR = 9.13; 95%CI: 1.47–56.80) compared to secondary school.

### Attitude and awareness towards biobanking

For what concerns attitude and awareness, the great majority of individuals (76.8%) chose the "pragmatic" response to motivate blood donation for research, and 89.9% were clearly aware of having been asked for additional blood for future use. The multivariate analysis did not show any differences between "ITR-enrolled" and "volunteer" subjects regarding these latter dimensions, but indicated that females had a greater awareness (OR = 8.77; 95%CI: 1.16–66.15).

[see Additional file 2]

Moreover, we applied the classical twin concordance analysis to gain insights into possible genetic and environmental effects on the "pragmatic" attitude to donation. This analysis involved 44 pairs where at least one twin chose the "pragmatic" response. Out of them, 24 MZ and 8 DZ pairs were concordant, while 6 MZ and 6 DZ pairs were discordant. Pairwise concordance rates were 0.80 (95%CI: 0.66–0.94) and 0.57 (95%CI: 0.31–0.83) in MZ and DZ pairs, respectively. The MZ vs DZ difference was not statistically significant, probably due to the low power of the study. However, the substantially higher concordance rate in MZ compared to DZ pairs was compatible with possible genetic influences on the expression of this trait.

## Discussion

On the international landscape, there are few investigations concerning issues such as motivation and willingness to donate for scientific research purposes. One study on motivation to participation in a large genetic-epidemiological research provided results which explain participation within an altruistic framework [17]. One population-based study [18] on consent for blood storing showed that a very high percentage of former participants to a WHO project asserted their willingness to contribute to future research, being favourable to the use of their blood samples collected many years previously. Other studies [19,20] variously confirmed high degree of trust in genetic research and comfort for blood donation. Yet, to our knowledge, there is not enough work that investigates understanding of studies' aims and method as well as attitude of participants, including healthy groups, towards biobanking and related research [21].

The present survey has to be considered explorative of these quite unknown phenomena, particularly in the Italian research context. Twins are a subgroup of the general population and a very powerful model for many genetic-epidemiological studies. Furthermore, twins do not differ from singletons for a number of traits including socio-demographic features and health outcomes [22,23]. For these reasons, our results can be of wider interest and offer suggestions regarding larger groups of population.

The first result is the higher survey response rate of volunteers compared to that of the ITR-enrolled individuals. The survey shows a low level of understanding of study aim of the overall sample and a significantly higher probability of understanding among the "volunteer" subjects compared to the "ITR-enrolled" ones. Age did not account for differences in understanding, but its effect might be masked by the fact that the two groups are quite distinct for age, being the "ITR-enrolled" individuals in their twenties and the "volunteer" ones in their forties, with only one volunteer in the same age range of ITR-enrolled. Hence, further investigation is necessary to disentangle the contributions of age and modality of recruitment. Moreover, no difference in understanding emerged in terms of elapsed time between Euroclot examination and response to the present survey. All the above results suggest the hypothesis that detailed information regarding objectives and methodology of a study may be of little interest to "ITR-enrolled" participants, perhaps because of their younger age or their approach to research. In this context, spontaneity in participation, not filtered by previous enrolment, might have had an impact on understanding. This is in accordance with a general interest of donors in research often described at international level [24] and with what Hoeyer [25] described, in the framework of population-based studies, as the process of a "surrogate decision" given by participants, that is likely to represent the will to rely on researchers and to entrust them to direct investigations towards specific objectives. Nevertheless, it cannot be excluded that a certain degree of failure in the communicative process during the informative consent procedures contributed to the generally poor understanding of aim, calling into question the content of the information conveyed, as well as the simplicity of expression and vocabulary [26]. It is also possible that communication problems had a different impact on "ITR-enrolled" and "volunteer" subjects. Furthermore, higher educational levels were expectedly found to be associated with better understanding, indicating that interpretation skills of individuals might play an important role in understanding [27]. Yet, differences between "ITR-enrolled" and "volunteer" participants remain and seem to be consistent with the hypothesis mentioned above of different "research participation behaviours" of the two groups.

Responses given to the second dimension investigated show a prevailing "pragmatic" motivation to donate for research. Our "construct" does not embrace the theory of moral judgement, developed in the philosophical and ethical field [28]; it was meant to describe socially relevant preferences and practical motivation of lay, healthy subjects to participate and donate for research, especially where direct benefits are not in the background. It is quite interesting to record this tendency compared to that of spontaneity and that of a moral duty. Population biobanking activities, which need large groups of healthy individuals, are quite new in our country and this emerging attitude, if confirmed by larger studies, will give advice about policies on research and biobanks. Pragmatic attitude might be speaking the language of trust, but also, in the long run, that of more cooperation and communication among the various stakeholders and lastly that of an increased social control.

Moreover, as twins are a valuable tool to investigate the relative role of genes and environment in determining human traits, a twin concordance analysis of the prevailing "pragmatic" attitude to donation for research suggested possible genetic influences on the expression of this behavioural trait. Future studies on larger twin samples might help to clarify whether genetic factors are of importance in determining inter-individual differences in what we regarded as a pragmatically-driven compliance with research donation. This question is also reasonable considering the substantial evidence of neurobiological mechanisms governing social judgments [29].

The appreciable donors' consciousness emerging in this study is also noteworthy; this is in agreement with a previous study [30] and in contrast with others [26], all of them relying on different designs. The high level awareness for donation, in particular for females, together with the "pragmatic" attitude let envisage the need of lay people to contribute to research in a sort of balanced relationship: competence, skills, financial resources from institutions on one side, participation and donation of bio-material from individuals on the other.

All this has to be considered within a social and cultural framework [31]. In Italy, attention and concern for privacy issues, even if originated from different grounds, together with a still worldwide dominant idea of DNA and genes as "threatening personal information" [32], might play an important role in determining a high level of consciousness and attention to donation for research. It would be important, in a second step, to ascertain whether awareness means a real concern about the destiny of the biological material, or represents an indirect concern for privacy or confidentiality issues [25,33], a need to "keep in contact" with the research groups and their activities.

The small sample size did not allow us to fully ascertain the role of socio-demographic variables, such as age, gender, education and modality of recruitment in the investigated dimensions. Another limitation of this survey regards the choice of "closed" response categories for the question on attitude that might have somewhat forced individuals' responses, leading to the exclusion of other motivations for donation.

## Conclusion

The survey adds information about comprehension and motivation of healthy research participants, which is worth being further investigated. Level of understanding of aims and methods of a specific research project seems to vary in relation to modalities of approaching research; most of the participants are well aware of having been asked to donate blood for biobanking activities, and seem to be motivated by a "pragmatic" attitude to contribute to research.

Given that research on common diseases is becoming largely dependent on population surveys and biobanking, it is necessary to promote a public debate on these issues [34]. Suggestions that people may have a "duty" to participate in research are not new on the international scenario [35], especially where risks are considered low for participants; even if the "dilemma" between respect of autonomy and respect of solidarity cannot be completely solved, a balance should be continuously pursued.

## Abbreviations

ITR: Italian Twin Registry; MZ: Monozygotic; DZ: Dizygotic; OR: odds ratio; 95% CI: 95% confidence interval.

## Competing interests

The authors declare that they have no competing interests.

## Authors' contributions

VT conceived and designed the study, analyzed and interpreted the data, drafted the manuscript and supervised the study; CF performed statistical analysis, interpreted and analyzed data, drafted the manuscript and supervised the study; LN conceived and designed the study, analyzed and interpreted data, critically revised the manuscript, supervised the study; CDI acquired the data, critically revised the manuscript and provided administrative and technical support; LG conceived and designed the study, critically revised the manuscript and provided administrative and technical support; SB performed statistical analysis, interpreted and analyzed data and critically revised the manuscript; MAS conceived and designed the study, critically revised the manuscript, supervised the study and obtained funding. All authors read and approved the final manuscript.

## Pre-publication history

The pre-publication history for this paper can be accessed here:



## Supplementary Material

Additional file 1 **Percentage distributions of understanding, attitude and awareness, by sex, education, and modality of recruitment**. The data provided (absolute and percentage values) describe the individuals enrolled and the frequency distributions of their replies to the questionnaire. Click here for file

Additional file 2 **Factors associated with understanding, attitude and awareness, according to multiple logistic regression models**. the data provided represent the statistical analysis of the probability of specific replies according to different variables. Click here for file

## References

[B1] Capron AM (1991). Protection of research subjects: do special rules apply in epidemiology?. J Clin Epidemiol.

[B2] Feinleib M (1991). The epidemiologist's responsibilities to study participants. J Clin Epidemiol.

[B3] Peltonen L (2007). Old suspect found guilty The first genome profile of Multiple Sclerosis. N Engl J Med.

[B4] Williams G, Schroeder D (2004). Human genetic banking: altruism, benefit and consent. New Genet Soc.

[B5] Laurie G (2008). Evidence of support for biobanking practices. BMJ.

[B6] Fagnani C, Brescianini S, Cotichini R, D'Ippolito C, Dukic T, Giannantonio L, Medda E, Nisticò L, Patriarca V, Pulciani S (2006). The Italian Twin Register: New cohorts and tools, current projects and future perspectives of a developing resource. Twin Res Hum Genet.

[B7] Toccaceli V, Nisticò L (2006). Promozione di una banca biologica per il Progetto Europeo "GenomEUtwin": riflessioni etiche e tutela della privacy nella conservazione di tessuti umani per la ricerca. Rapporto ISTISAN.

[B8] Sgreccia E (2000). La bioetica e i suoi principi. Manuale di Bioetica.

[B9] Barilan YM (2004). Towards a dialogue between utilitarianism and medicine. Med Health Care Philos.

[B10] Hoeyer K (2003). "Science is really needed – that's all I know": informed consent and the non-verbal practices of collecting blood for genetic research in Northern Sweden. New Genet Soc.

[B11] Kyvik KO, Green A, Beck-Nielsen H (1995). The New Danish Twin Register: establishment and analysis of twinning rates. Int J Epidemiol.

[B12] Flory J, Emanuel E (2004). Interventions to improve research participants' understanding in informed consent for research. A systematic review. JAMA.

[B13] Italian Authority for personal data protection: Authorization to genetic data treatment, 22nd February 2007. http://www.garanteprivacy.it/garante/navig/jsp/index.jsp?folderpath=Normativa%2FItaliana%2FAutorizzazioni+del+Garante%2F2007.

[B14] Italian Legislative Decree n. 196, 30th June DataProtectionCode2003_Consolidated Text in Force. http://www.garanteprivacy.it/garante/navig/jsp/index.jsp?folderpath=Normativa%2FItaliana%2FIl+Codice+in+materia+di+protezione+dei+dati+personali.

[B15] Boomsma D, Busjahn A, Peltonen L (2002). Classical twin studies and beyond. Nat Rev Genet.

[B16] Neale MC, Cardon LR (1992). Methodology for genetic studies of twins and families.

[B17] Treloar SA, Morley KI, Taylor SD, Hall WD (2007). Why do they do it? A pilot study towards understanding participant motivation and experience in a large genetic epidemiological study of endometriosis. Community Genet.

[B18] Stegmayr B, Asplund K (2002). Informed consent for genetic research on blood stored for more than a decade: a population based study. BMJ.

[B19] Lindblad KA, Ring L, Viberth E, Hanson MG (2006). Genetic research and donation of tissue samples to biobanks. What do potential sample donors in the Swedish general public think?. Eur J Public Health.

[B20] Khalil SS, Silverman HJ, Raafat M, El-Kamary S, El-Setouhy M (2007). Attitudes, understanding and concerns regarding medical research amongst Egyptians: A qualitative pilot study. BMC Med Ethics.

[B21] Joseph JW, Neidich AB, Ober C, Ross LF (2008). Empirical data about women's attitudes toward a biobank focused on pregnancy outcomes. Am J Med Genet A.

[B22] Kendler KS, Martin NG, Heath AC, Eaves LJ (1995). Self-report psychiatric symptoms in twins and their non-twin relatives: Are twins different?. Am J Med Genet.

[B23] Andrew T, Hart DJ, Snieder H, de Lange M, Spector TD, MacGregor AJ (2001). Are twins and singletons comparable? A study of disease-related and lifestyle characteristics in adult women. Twin Res.

[B24] Hansson MG, Dillner J, Bartram CR, Carlson JA, Helgesson G (2006). Should donors be allowed to give broad consent to future biobank research. Lancet Oncol.

[B25] Hoeyer K (2004). Informed consent and biobanks: a population-based study of attitudes towards tissue donation for genetic research. Scand J Public Health.

[B26] Moutel G, de Montgolfier S, Meningaud JP, Hervé C (2001). Bio-libraries and DNA storage: assessment of patient perception of information. Med Law.

[B27] Lukoschek P, Fazzari M, Marants P (2003). Patient and physician factors predict patients' comprehension of health information. Patient Educ Couns.

[B28] Frey RG (2003). Utilitarianism and bioethics. Post SG Encyclopedia of bioethics.

[B29] Moll J, de Olivera-Souza R (2007). Moral Judgments, emotions and the utilitarian brain. Trends Cog Sci.

[B30] Nakayama T, Muto K, Yoshiike N, Yokoyama T (1999). Awareness and Motivation of Japanese Donors of Blood for Research. Am J Pub Health.

[B31] Haimes E, Whong-Barr M, Knoppers BM (2003). Competing perspectives on reasons for participation and non-participation in the North Cumbria Community Genetics Project. Populations and Genetics: Legal Socio-Ethical Perspectives.

[B32] International Declaration on Human Genetic Data. (UNESCO). http://portal.unesco.org/shs/en/ev.php-URL_ID=1882&URL_DO=DO_TOPIC&URL_SECTION=201.html.

[B33] Hoeyer K, Olofsson BO, Mjörndal T, Lynöe N (2005). The ethics of Research using Biobanks. Reason to question the importance attributed to informed consent. Arch Intern Med.

[B34] Beskow LM, Dean E (2008). Informed consent for biorepositories: assessing prospective participants understanding and opinions. Cancer Epidemiol Biomarkers Prev.

[B35] Chadwick R, Berg K (2001). Solidarity and equity: new ethical frameworks for genetic databases. Nat Rev Genet.

