# Supplementary material for: Research understanding, attitude and awareness towards biobanking: a survey among Italian twin participants to a genetic epidemiological study
Source: BMC Med Ethics. 2009 Jun 16;10:4. doi: 10.1186/1472-6939-10-4 (PMC2703640; doi:10.1186/1472-6939-10-4)
Supplement: Additional file 1 — Percentage distributions of understanding, attitude and awareness, by sex, education, and modality of recruitment. The data provided (absolute and percentage values) describe the individuals enrolled and the frequency distributions of their replies to the questionnaire. [file 1472-6939-10-4-S1.doc]

**Table 1**

Percentage distributions of understanding, attitude and awareness, by sex, education, and modality of recruitment

|  | **Understanding** | | | | | | | | **Attitude** | | | | **Awareness** | | |
| --- | --- | --- | --- | --- | --- | --- | --- | --- | --- | --- | --- | --- | --- | --- | --- |
|  | Aim | | | | Method | | | |  | | | |  | | |
|  | C | F | DKorDR | Total | C | F | DKorDR | Total | M | P | S | Total | Yes | DR | Total |
| **Sex** |  |  |  |  |  |  |  |  |  |  |  |  |  |  |  |
| Males (n = 26) | **34.6** | **42.3** | **23.1** | **100** | **84.6** | **3.8** | **11.5** | **100** | **3.8** | **84.6** | **11.5** | **100** | **73.1** | **26.9** | **100** |
|  | (9) | (11) | (6) |  | (22) | (1) | (3) |  | (1) | (22) | (3) |  | (19) | (7) |  |
| Females (n = 73) | **32.9** | **57.5** | **9.6** | **100** | **72.6** | **16.4** | **11.0** | **100** | **13.7** | **74.0** | **12.3** | **100** | **95.9** | **4.1** | **100** |
|  | (24) | (42) | (7) |  | (53) | (12) | (8) |  | (10) | (54) | (9) |  | (70) | (3) |  |
| **Education*** |  |  |  |  |  |  |  |  |  |  |  |  |  |  |  |
| Secondary school (n = 11) | **18.2** | **63.6** | **18.2** | **100** | **72.7** | **9.1** | **18.2** | **100** | **18.2** | **63.6** | **18.2** | **100** | **90.9** | **9.1** | **100** |
|  | (2) | (7) | (2) |  | (8) | (1) | (2) |  | (2) | (7) | (2) |  | (10) | (1) |  |
| High school (n = 60) | **31.7** | **53.3** | **15.0** | **100** | **73.3** | **15.0** | **11.7** | **100** | **10.0** | **76.7** | **13.3** | **100** | **88.3** | **11.7** | **100** |
|  | (19) | (32) | (9) |  | (44) | (9) | (7) |  | (6) | (46) | (8) |  | (53) | (7) |  |
| College (n = 28) | **42.9** | **50.0** | **7.1** | **100** | **82.1** | **10.7** | **7.1** | **100** | **10.7** | **82.1** | **7.1** | **100** | **92.9** | **7.1** | **100** |
|  | (12) | (14) | (2) |  | (23) | (3) | (2) |  | (3) | (23) | (2) |  | (26) | (2) |  |
| **Modality of Euroclot recruitment** |  |  |  |  |  |  |  |  |  |  |  |  |  |  |  |
| “ITR-enrolled” (n = 61) | **16.4** | **70.5** | **13.1** | **100** | **68.9** | **18.0** | **13.1** | **100** | **6.6** | **80.3** | **13.1** | **100** | **88.5** | **11.5** | **100** |
|  | (10) | (43) | (8) |  | (42) | (11) | (8) |  | (4) | (49) | (8) |  | (54) | (7) |  |
| “Volunteers” (n = 38) | **60.5** | **26.3** | **13.2** | **100** | **86.8** | **5.3** | **7.9** | **100** | **18.4** | **71.1** | **10.5** | **100** | **92.1** | **7.9** | **100** |
|  | (23) | (10) | (5) |  | (33) | (2) | (3) |  | (7) | (27) | (4) |  | (35) | (3) |  |
| **Total sample (n = 99)** | **33.3** | **53.5** | **13.1** | **100** | **75.8** | **13.1** | **11.1** | **100** | **11.1** | **76.8** | **12.1** | **100** | **89.9** | **10.1** | **100** |
|  | (33) | (53) | (13) |  | (75) | (13) | (11) |  | (11) | (76) | (12) |  | (89) | (10) |  |

Abbreviations: C: correct; F, false; DK or DR: I don’t know/remember; M: moral duty; P: pragmatism; S: spontaneity.

In parentheses are numbers of subjects

* number of years of education corresponding to each level: secondary school=8 years, high school=13 years, college=17-19 years
