# Supplementary material for: Research understanding, attitude and awareness towards biobanking: a survey among Italian twin participants to a genetic epidemiological study
Source: BMC Med Ethics. 2009 Jun 16;10:4. doi: 10.1186/1472-6939-10-4 (PMC2703640; doi:10.1186/1472-6939-10-4)
Supplement: Additional file 2 — Factors associated with understanding, attitude and awareness, according to multiple logistic regression models. the data provided represent the statistical analysis of the probability of specific replies according to different variables. [file 1472-6939-10-4-S2.doc]

**Table 2**

Factors associated with understanding, attitude and awareness, according to multiple logistic regression models

|  | **Understanding** | | **Attitude**  (pragmatism vs others) | **Awareness** |
| --- | --- | --- | --- | --- |
|  | Aim | Method |  |  |
|  | OR (95% CI)^a^ | OR (95% CI)^a^ | OR (95% CI)^a^ | OR (95% CI)^a^ |
| **Age** (years) | 0.99 (0.94-1.05) | 0.94 (0.87-1.02) | 1.02 (0.94-1.10) | 1.06 (0.99-1.14) |
| **Sex** |  |  |  |  |
| Males | 1 | 1 | 1 | 1 |
| Females | 0.64 (0.17-2.35) | 0.38 (0.10-1.53) | 0.26 (0.06-1.13) | 8.77 (1.16-66.15) |
| **Education** |  |  |  |  |
| Secondary school | 1 | 1 | 1 | 1 |
| High school | 6.06 (0.95-38.91) | 2.41 (0.43-13.40) | 2.32 (0.40-13.58) | 0.44 (0.02-12.38) |
| College | 9.13 (1.47-56.80) | 3.53 (0.59-20.94) | 4.89 (0.53-45.38) | 0.67 (0.01-33.47) |
| **Modality of Euroclot recruitment** |  |  |  |  |
| ITR-enrolled | 1 | 1 | 1 | 1 |
| volunteers | 8.23 (1.28-52.81) | 11.41 (1.10-118.38) | 0.40 (0.07-2.30) | 0.39 (0.03-4.90) |

Abbreviations: OR, odds ratio; 95% CI, 95% confidence interval.

^a^ 95% CI = Models were fitted to twins as individuals, and standard errors were adjusted for clustering on twin pairs. Odds Ratios were also

adjusted for elapsed time between Euroclot examination and response.
